# Supplementary material for: Analysis of the effect of the scorpion toxin AaH-II on action potential generation in the axon initial segment
Source: Sci Rep. 2024 Feb 29;14:4967. doi: 10.1038/s41598-024-55315-y (PMC10904771; doi:10.1038/s41598-024-55315-y)
Supplement: Supplementary file 1 — Supplementary Legends. [file 41598_2024_55315_MOESM1_ESM.docx]

***Analysis of the effect of the scorpion toxin AaH-II on action potential generation in the axon initial segment***

**Fatima Abbas**^1,2,5^, **Laila Ananda Blömer**^1,2,5^, **Hugo Millet**^2,3^, **Jérôme Montnach**^2,3^, **Michel De Waard**^2,3^, **Marco Canepari**^1,2,4,*^

**Supplementary Movie S1 Legend**

Colour scale movies reporting the normalised Ca^2+^ transients associated with action potentials in the axon initial segment under control conditions (top) and after local delivery of 7 nM of AaH-II. The somatic action potentials are illustrated on the right. AaH-II enhances Ca^2+^ influx mostly in the distal part of the axon initial segment.
